# Viral polymerase-host interaction analysis reveals that the association between avian NUP93 and PB1 promotes H5N6 avian influenza virus replication

**DOI:** 10.1128/spectrum.03120-24

**Published:** 2025-05-23

**Authors:** Lei Cao, Ting Wang, Peilei Ren, Chuxing Cheng, Haiying Mao, Xianfeng Hui, Xian Lin, Xiaotong Hu, Xiaomei Sun, Kun Huang, Meilin Jin

**Affiliations:** 1 State Key Laboratory of Agricultural Microbiology, Huazhong Agricultural University 47895, Wuhan, China; 2 College of Animal Medicine, Huazhong Agricultural University 47895, Wuhan, China; 3 Center for Cross-disciplinary Studies in Animal Epidemic and Public Health, Hubei Jiangxia Laboratory, Wuhan, China; University of Manitoba, Winnipeg, Manitoba, Canada

**Keywords:** H5N6 avian influenza virus, host protein, polymerase, PB1, NUP93

## Abstract

The H5N6 avian influenza virus, a highly pathogenic strain, poses a significant threat to poultry production and public health. The RNA-dependent RNA polymerase (comprising PB1, PB2, and PA proteins) and nucleoprotein of highly pathogenic avian influenza viruses interact with avian host proteins, influencing the efficiency of viral RNA synthesis and severity of infection. To comprehensively understand how H5N6 avian influenza virus interfaces with host cellular mechanisms during infection and replication, it is essential to identify which host proteins physically associate with viral polymerase proteins. In this study, affinity purification mass spectrometry was used to identify physical interactions between H5N6-JX polymerase proteins (PB1, PB2, PA, and nucleoprotein) and host proteins in chicken DF-1 cells. We identified 455 H5N6-chicken interacting proteins and successfully cloned 231 of these genes. Overexpression experiments revealed several host proteins involved in viral replication in DF-1 cells. Specifically, nine host genes were found to promote avian influenza virus proliferation, whereas 20 inhibited it. Furthermore, we demonstrated that avian NUP93 interacts with the viral PB1 protein, enhancing polymerase transcriptional activity and promoting viral proliferation. These findings provide a more comprehensive understanding of how host mechanisms are manipulated during H5N6 avian influenza viral infection and replication, providing insights into the mechanisms of avian influenza virus cross-species transmission.

**IMPORTANCE:**

The RNA-dependent interaction of RNA polymerase with avian host protein determines the efficiency of viral RNA synthesis and the severity of infection. However, the strain-specific interactions of the avian influenza virus (AIV) remain unclear. In this study, we identified 455 H5N6-chicken interacting proteins and successfully cloned 231 of them. Nine host genes that promote the proliferation of avian influenza virus and 20 host genes that inhibit the proliferation of avian influenza virus were identified through overexpression experiments. In addition, we demonstrated that avian NUP93 interacts with viral PB1 protein to enhance polymerase transcriptional activity and promote viral proliferation. This study contributes to a more comprehensive and detailed understanding of the molecular mechanisms of host utilization during the H5N6 highly pathogenic avian influenza virus infection and replication.

## INTRODUCTION

The influenza A virus (IAV) is a pathogen that causes zoonotic respiratory diseases, leading to 650,000 deaths annually and posing a significant threat to global public health (1, 2). IAV can cross species barriers to infect various animals, including bats, pigs, cattle, poultry, horses, and dogs, with aquatic birds as its natural hosts (3, 4). Several avian IAV strains, such as H5N1 and H7N9, have acquired the ability to infect humans, resulting in high fatality rates (5, 6). More recently, an outbreak of the H5N1 avian influenza subtype 2.3.4.4b affected more than 240 dairy herds, leading to at least four human infections in the United States (7, 8). This can result in spread among mammals, potentially causing lethal diseases (9).

IAV is an enveloped negative-sense RNA virus with eight RNA segments that encode at least 17 viral proteins (including the packaging signals of IAV) (10). Like most RNA viruses, the replication of influenza in cells depends on its polymerase complex, which consists of viral RNA (vRNA) segments associated with multiple viral nucleoproteins (NPs), including polymerase basic protein 2 (PB2), polymerase basic protein 1 (PB1), and polymerase acidic protein (PA) (11). After IAV enters the host cell, viral ribonucleoproteins (vRNPs) are released into the cytoplasm and imported into the nucleus for replication and transcription. The newly produced vRNPs are then exported from the nucleus and assembled with other viral proteins to produce progeny virions. During this process, the virus relies on host cellular functions to replicate, hijacking the host cell machinery and rewiring host factors for its own needs. Therefore, understanding the interactions between avian IAV and avian hosts is crucial for preventing and controlling cross-species transmission of avian influenza.

Numerous proteomic studies have employed affinity purification mass spectrometry to identify a range of human cellular host factors (12, 13) that interact with IAV proteins. Recent studies have identified avian host factors (12, 14) interacting with H5N1. However, the strain-specific interactions of the avian influenza virus (AIV) remain unclear, and the mechanism by which these interactions control host defense and viral infection has not been fully elucidated. In this study, to determine which host proteins physically interact with viral polymerase proteins, we applied affinity purification mass spectrometry (AP-MS) technology to reveal the host proteins, complexes, and pathways associated with polymerase during H5N6 AIV infection. Furthermore, we constructed a map of the interaction between viral polymerase and host genes. Additionally, 231 of the 455 host factors were successfully cloned, and the effect on influenza virus was evaluated. Among these novel factors, the nuclear pore complex protein 93 (NUP93) interacts with the influenza virus PB1 protein to facilitate the replication of H5N6 AIV by enhancing viral polymerase transcription activity.

## MATERIALS AND METHODS

### Cell culture and virus

Chicken embryonic fibroblast (DF-1) cells and Madin–Darby canine kidney (MDCK) cells were cultured in Dulbecco’s modified Eagle’s medium (DMEM) supplemented with 10% fetal bovine serum (PAN-Biotech) and 1% penicillin-streptomycin at 37°C in 5% CO_2_. The IAV strains used were A/duck/Hubei/WH18/2015 (H5N6-JX) (GenBank accession number KX652135)(15), A/chicken/Hubei/327/2004 (H5N1-DW), A/chicken/Guangxi/97/2017 (H7N9-GX), and H5N6-GFP recombinant virus. All viruses were amplified from 10-day-old specific pathogen-free embryonated eggs and stored in our laboratory. Experiments involving live viruses were performed in a biosafety level 3 laboratory (BSL3) at the Huazhong Agricultural University (HZAU). All the procedures were approved by the Institutional Biosafety Committee of HZAU.

### Plasmids and small interfering RNAs

The PB1, PB2, PA, and NP genes of H5N6-JX were cloned into the eukaryotic expression vector, pCAGGS-HA. Host genes were cloned into the pCMV-3xFlag vector. Polymerase chain reaction (PCR) primers were designed using Primer 5, and all plasmid constructs were confirmed by sequencing. siRNAs targeting chicken NUP93 were purchased from Tsingke (Beijing, China). Knockdown efficiency was examined by Western blotting. Primer and siRNA sequences are listed in Table S2 of the Supplemental Material.

### Antibodies

The antibodies used in this study were as follows: anti-NUP93 rabbit polyclonal antibody (Proteintech, 1:500), anti-HA and anti-GAPDH mouse monoclonal antibodies (Proteintech, 1:3,000), anti-Flag mouse monoclonal antibody (Sigma, 1:3,000), anti-IAV PB1 and NP rabbit polyclonal antibodies (GeneTex,1:3,000), and Alexa Fluor 488-conjugated AffiniPure goat anti-mouse (Proteintech, 1:500) and Alexa Fluor 594-conjugated AffiniPure goat anti-rabbit (Proteintech, 1:500) secondary antibodies.

### Immunoprecipitation

For exogenous protein immunoprecipitation, DF-1 cells were transfected with the indicated plasmids using Lipofectamine 2000 (Invitrogen). After 24 h of transfection, the cells were lysed in Western and IP cell lysis buffer (Beyotime) containing a protease inhibitor cocktail (MCE, #HY-K0010) on ice. Subsequently, the partially cleared cell lysate was used in the immunoprecipitation experiment by incubating it with anti-HA (MCE, #HY-K0201) magnetic beads at 4°C overnight, while the remaining cell lysate was used as an immunoprecipitation input.

Endogenous immunoprecipitation was performed using protein A/G agarose (Santa Cruz, #SC-2003). According to the manufacturer’s instructions, DF-1 cells were mock-infected or infected with the H5N6-JX virus (multiplicity of infection [MOI] = 1) for 12 h. The cells were then lysed, and the immunoprecipitation assay was conducted using anti-PB1 anti-NP, or anti-NUP93 antibodies (10 µg/mL each), with rabbit IgG antibody as a control. The cell lysate and antibody mixtures were incubated at 4°C overnight. The next day, pretreated protein A/G agarose was added to the mixture and incubated at 4°C for another 2 h with rotation. Immunoprecipitated proteins and input cell lysates were analyzed using Western blotting.

### Western blotting

Cells were lysed in Western and IP cell lysis buffer (Beyotime, P0013J) after two PBS washes. Proteins were quantified by standardization (20 µg total cellular protein/well). Each protein sample was separated using 10%–12% sodium dodecyl-sulfate polyacrylamide gel electrophoresis (SDS-PAGE) and transferred onto a polyvinylidene difluoride membrane. Subsequently, the membrane was immersed in 5% milk for 1 h at ambient temperature and incubated with primary and secondary antibodies successively. Finally, images were captured using an ECL detection system (Tannon, Beijing, China). The integrated OD (IOD) of NUP93, NP, and GAPDH blots was determined using Image-Pro Plus software (Media Cybernetics, Rockville, MD), and the relative IOD of NUP93 and NP was calculated by dividing the IOD of NUP93 or NP/IOD GAPDH. All the relative IOD values were normalized to that of lane 1 and are shown below each blot. All experiments were repeated at least three times, and representative results are shown.

### Mass spectrometry analysis

To identify vRNP-associated host factors, we performed immunoprecipitation mass spectrometry (IP/MS). DF-1 cells were seeded onto six well plates followed by transfection or infection over indicated times. The cells were then lysed and subjected to immunoprecipitation, Western blotting, and silver staining. Protein bands were excised and subjected to tryptic digestion. After reduction and alkylation, trypsin (mass ratio 1:50) was added, and the mixture was hydrolyzed at 37°C for 20 h. After desalination, the enzymatic hydrolysis product was lyophilized and redissolved in 0.1% formic acid solution. Tandem mass spectrometry (MS/MS) signals were processed against the UniProt Gallus protein database using the Mascot 2.2 algorithm with the following parameters: variable modifications, oxidation (Met), N-acetylation, and pyroglutamination (Gln); maximum missed cleavages, 2; peptide mass tolerance, 100 ppm; and MS/MS tolerance, 0.5 Da. The criterion used for protein identification was based on at least one MS/MS data signal with Mascot scores exceeding the threshold (*P* < 0.05).

### Bioinformatics analysis

Functional annotation and classification of all identified proteins were conducted using clusterProfiler V4.10.1, which included both Gene Ontology (GO) and Kyoto Encyclopedia of Genes and Genomes (KEGG) functional enrichment analyses. Protein-protein interaction (PPI) networks were constructed and visualized using Cytoscape software.

### Indirect immunofluorescence imaging

DF-1 cells were grown on coverslips and transfected with the indicated plasmids using Lipofectamine 2000 for 24 h or with H5N6-JX (MOI = 10) for 12 h. Cells were fixed in 4% paraformaldehyde for 30 min at room temperature and permeabilized with 0.1% Triton X-100 (Sigma-Aldrich) for 15 min. The cells were incubated with primary antibodies for 2 h and then with the appropriate Alexa Fluor-conjugated secondary antibody (Proteintech, 1:500) for 1 h before the nuclei were stained with DAPI (Beyotime, 1:1,000) for 15 min at room temperature. Finally, the stained samples were visualized under a confocal microscope (LSM 880; Zeiss, Oberkochen, Germany).

### Minigenome assay

DF-1 cells were co-transfected with the indicated host genes along with pcDNA3.1-PB1, pcDNA3.1-PB2, pcDNA3.1-PA, pcDNA3.1-NP, and pPolI-AVI, which contains the firefly luciferase gene flanked by the noncoding regions of the avian influenza nonstructural gene segment, as well as a *Renilla* luciferase expression construct, for normalization. After 24 h post-transfection, relative polymerase activity (firefly normalized to *Renilla*) was measured using a dual-luciferase reporter assay kit (Promega, USA). *Renilla* luciferase activity was used as an internal control to normalize transfection efficiency.

### Transfection and virus titration

Plasmid and siRNA transfections were performed using Lipofectamine 2000 (Invitrogen, Carlsbad, CA, USA) in all the cells maintained in Opti-MEM. The culture medium was replaced with fresh medium supplemented with 10% FBS at 6 h post-transfection.

For IAV titration, viral supernatants were harvested at the indicated time points, serially diluted in DMEM, and adsorbed onto MDCK cell monolayers seeded onto 96-well plates. After 1 h of incubation, the supernatants were discarded, and the cells were washed twice with PBS. The plates were incubated at 37°C for 72 h. Virus titers were determined by calculating the log_10_ TCID_50_/mL in MDCK cells using the Spearman–Karber method (16).

### Flow cytometry analysis

Cells were seeded onto 12-well plates and transfected with plasmids to approximately 80% confluence (using empty vector as a negative control, with three replicates). At 24 h post-transfection, the cells were infected with H5N6-GFP influenza virus at an MOI of 0.01. After 24 h of incubation, fluorescence was observed using an inverted fluorescence microscope, revealing noticeable differences in fluorescence intensity between the experimental and control groups. Following the above treatment, the culture medium was discarded, and the cells were washed 2–3 times with PBS. The cells were then trypsinized and centrifuged at 1,000 rpm for 2 min. After discarding the supernatant, 4% paraformaldehyde was added to resuspend and fix the cells. Finally, the average fluorescence intensity was analyzed using a flow cytometer (Beckman, USA).

### Statistical analysis

Data from three independent experiments are expressed as means ± standard deviation (SD). Statistically significant analysis findings were based on *P* values using an unpaired two-tailed Student’s *t* test or two-way ANOVA (ns, not significant; *P  <  0.05; **P  <  0.01; ***P  <  0.001).

## RESULTS

### Identification of host proteins associated with vRNP complex and involved in influenza viral replication

**Fig 1:**
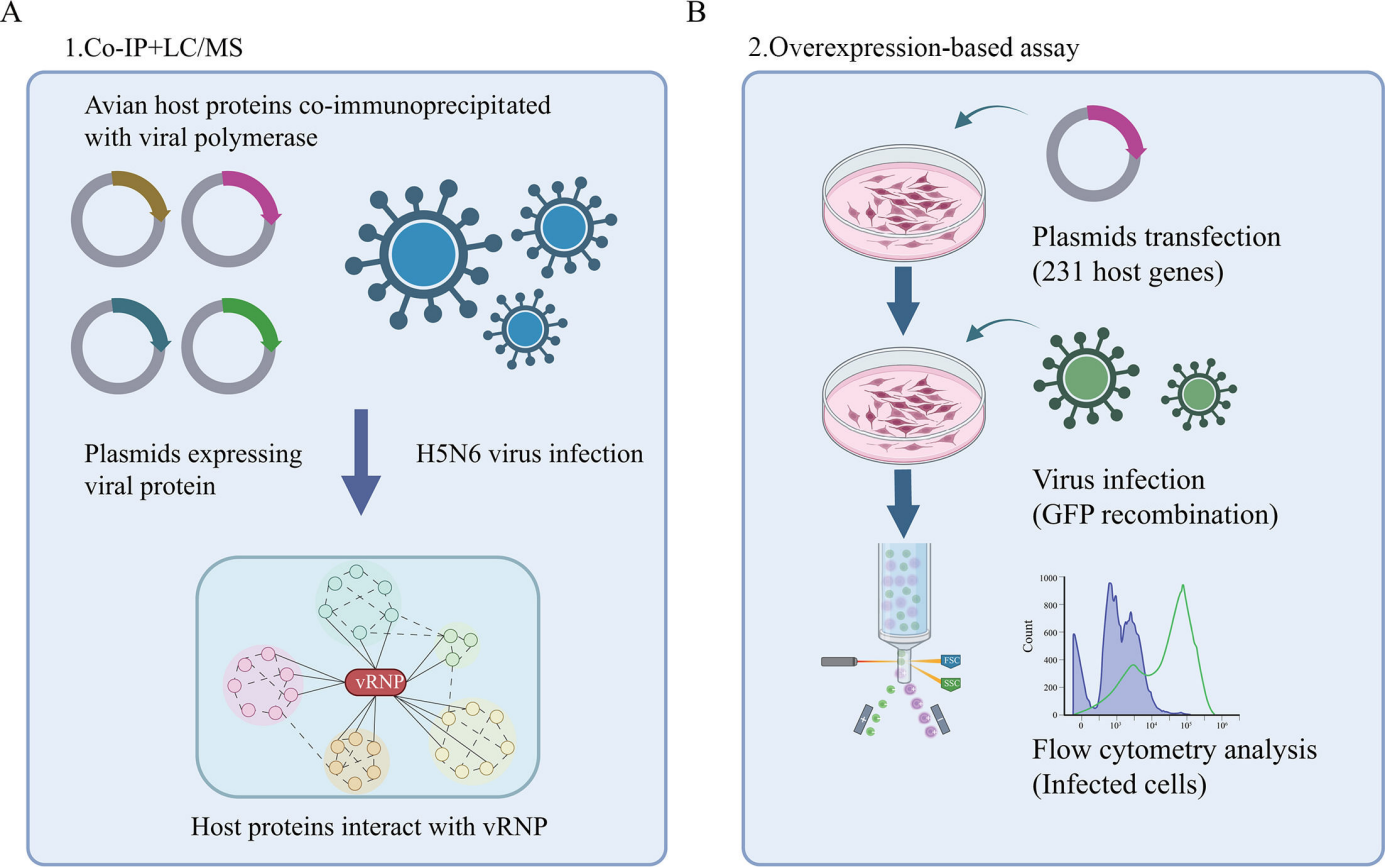
Overview of a systematic study to identify the physical interactions and functions between avian host and viral polymerase in AIV replication. (**A and B**) Schematic diagram illustrating the identification of host proteins that coprecipitated with avian influenza A viral polymerase proteins, affecting viral replication (created with BioRender.com).

Understanding the interactions between the IAV vRNP and its specific avian host partner can provide new insights into the mechanisms by which viruses promote their intracellular life cycle. To identify host factors that may participate in the pathogenesis of avian influenza infection, we first attempted to identify factors that are directly manipulated through endogenous and exogenous physical associations with viral polymerase proteins. Before conducting the aforementioned immunoprecipitation experiments, we first infected DF-1 cells with a recombinant GFP-expressing H5N6 virus using different infection doses. After 24 h, fluorescence observation revealed that an infection dose of 0.01 MOI was able to infect most cells while causing minimal cytopathic effects (Fig. S1A). Subsequently, we evaluated the transfection efficiency of Lipofectamine 2000 reagent in DF-1 cells by transfecting a GFP-expressing plasmid. The results demonstrated that a transfection ratio of 1:1 (plasmid to Lipofectamine 2000) achieved high transfection efficiency with low cytotoxicity (Fig. S1B). Therefore, these conditions were adopted for further experiments. Then, DF-1 cells were transfected with four HA-labeled IAV (A/Duck/Hubei/WH18/2015, H5N6-JX) viral polymerase plasmids (including PB2, PB1, PA, and NP) or directly infected with H5N6-JX influenza viruses at an MOI of 0.1. Immunoprecipitation was then performed using anti-HA, anti-PB1, anti-NP, and control IgG antibodies (Fig. 1A). Immunoprecipitated samples from plasmid-transfected or virus-infected cells were separated by SDS-PAGE for mass spectrometry analysis. Mass spectrometry analysis of the co-precipitated proteins identified 455 host proteins in total, and among these, 71, 59, 215, 151, and 130 proteins co-precipitated with the exogenous viral PA, PB1, PB2, NP proteins, and endogenous PB1 and NP (representing viral polymerase complexes) (17), respectively (Table S1). The coprecipitated host proteins may be specific binding partners of influenza viral proteins with essential or non-essential functions in the viral life cycle. Alternatively, they may be non-specific, that is, false-positive binding partners resulting from experimental artifacts, such as the overexpression of viral proteins in our assay and/or the absence of other viral components. Therefore, to identify host factors that are specifically involved in viral replication, we transfected DF-1 cells with plasmids encoding each of the 455 candidate host genes (231 genes were cloned successfully, Table S2). We then infected them with H5N6-GFP virus at 24 h post-transfection, followed by flow cytometry analysis of the viral load at 24 h post-infection (Fig. 1B).

### H5N6 AIV-chicken interactome

**Fig 2:**
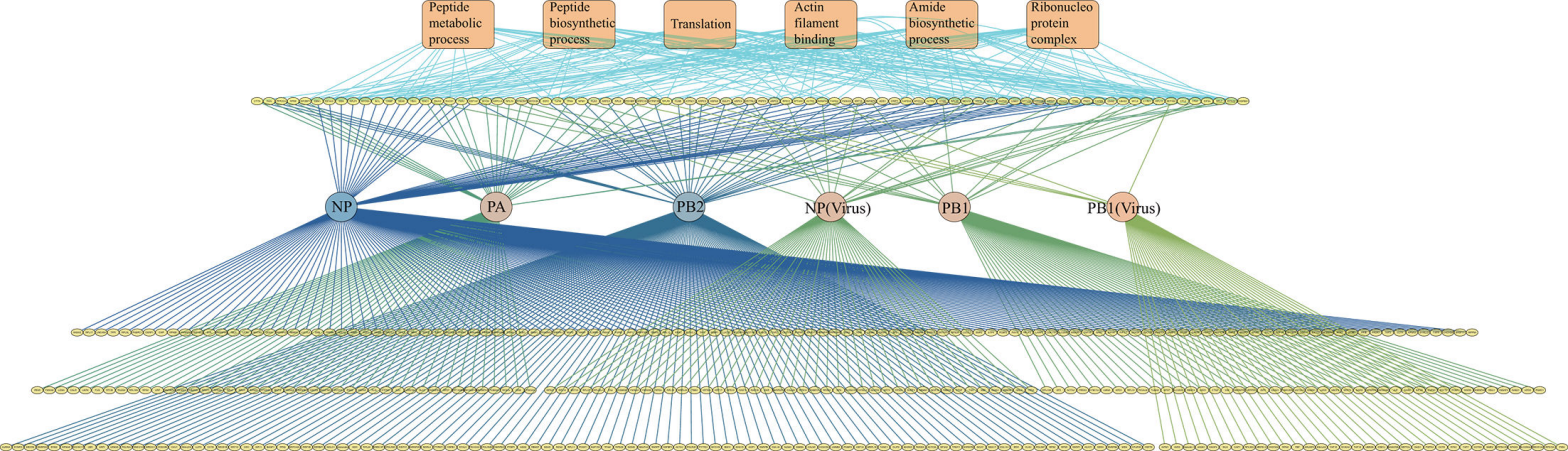
Network of avian host-avian influenza viral polymerase protein interactions. Interactions among the viral polymerase proteins and 455 host factors were visualized using Cytoscape (http://cytoscape. org/). Also, the Gene Ontology (GO) annotation of the host proteins interacting with the H5N6 polymerase complex is also shown. The network image is fully zoomable on the monitor. See Tables S1 and S3.

Next, we used Cytoscape to create a network diagram that included 455 interactions between AIV proteins and the H5N6-JX polymerase complex (Fig. 2). The circular nodes in the third row of the network represent the PB1, PB2, PA, and NP proteins of AIV transfected into DF-1 cells and the viral endogenous PB1 and NP proteins. The circular nodes in the second and fourth to sixth rows represent 455 host proteins from DF-1 cells (yellow). Meanwhile, the deeper the color of the lines originating from the viral proteins, the greater the number of host proteins interacting with the viral proteins (light green indicates the fewest interactions, while dark blue indicates the most interactions). These represent host proteins that interact with only one or more viral proteins. The network structure indicates that a large proportion of the host proteins that interact with this specific influenza virus not only influence the virus but also likely have broader biological functions and interact with multiple viral polymerase proteins. Next, we used ClusterProfiler to generate a GO annotation of the host proteins that interacted with the H5N6 polymerase complex (top orange block). In this network, the enriched host proteins mainly included peptide metabolic processes, peptide biosynthetic processes, translation, actin filament binding, amide biosynthetic processes, and vRNP complexes, which are closely related to the biological functions of influenza virus polymerase (18–20). Next, we compared our data with other data sets related to IAV infection, including previously published H5N1 AIV and chicken PPIs (14, 21) as well as host proteins associated with H1N1 IAV replication identified during CRISPR/Cas9 or AP/MS screening (12, 13, 22–25). Our analysis revealed that among the 455 host-interacting proteins identified in this study, 421 had been previously linked to IAV infection in both chickens and mammals. Notably, this screening identified 34 novel genes, thereby expanding the repertoire of host factors potentially involved in the life cycle of AIVs (Table S3). Additionally, using the Search Tool for the Retrieval of Interacting Genes/Proteins (STRING) to analyze all H5N6 AIV-host protein interactions (26), it is clear that many host-interacting proteins also interacted with other host proteins, and some even formed protein complexes with viral proteins (Fig. S2).

### Functional analysis of AIV vRNP complex interacting with the host proteins

**Fig 3:**
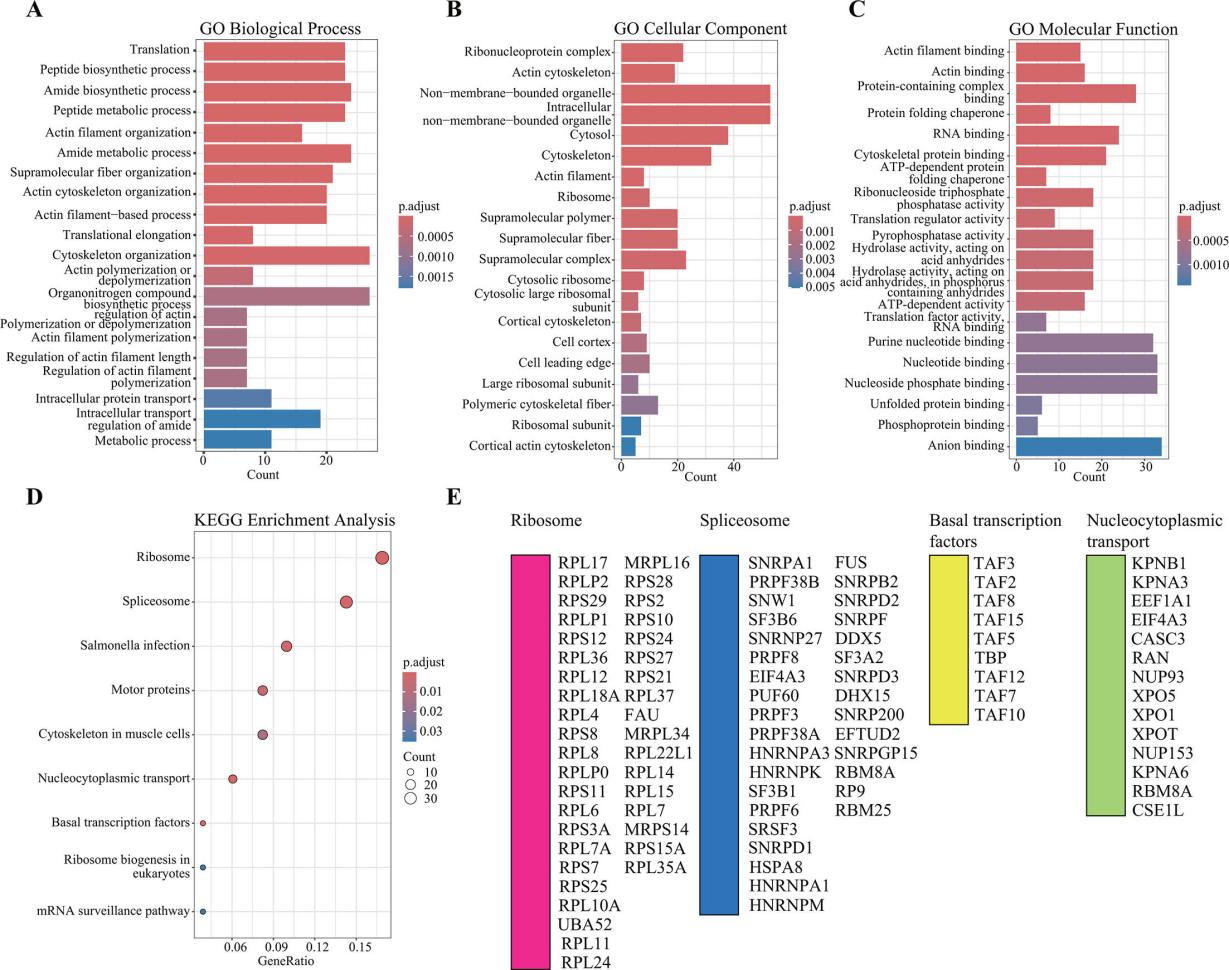
Pathway analysis of cellular proteins interacting with vRNP based on GO and KEGG. (**A–C**) GO annotation was analyzed using clusterProfiler, with the percentage of each GO component shown. (**A**) Biological process. (**B**) Cellular components. (**C**) Molecular function. (**D**) Classification of the enriched KEGG pathways of the identified proteins. (**E**) Name of the identified proteins related to the top four KEGG pathways classifications.

To achieve a comprehensive understanding of the functional characterization of the interaction networks associated with the viral vRNP complex, we employed ClusterProfiler for advanced functional annotation and classification of the 455 identified proteins, including GO and KEGG functional enrichment analyses. As shown in Fig. 3, the GO analysis yielded three primary annotation categories: biological processes, cellular components, and molecular functions. Furthermore, subclasses related to translation, peptide biosynthetic processes, and amide biosynthetic processes were significantly enriched in the biological process category (Fig. 3A). The most enriched subclasses of cellular components included the vRNP complex, actin cytoskeleton, and non-membrane-bound organelles (Fig. 3B). Additionally, molecular function analysis indicated that actin filaments, actin, and protein-containing complex binding were significantly enriched (Fig. 3C). A more detailed summary of the GO annotations of the identified proteins is provided in Table S4. Moreover, KEGG pathway analysis revealed 95 pathways linked to 301 infected networks (Table S5); pathways associated with ribosomes, spliceosomes, basal transcription factors, and nucleocytoplasmic transport were significantly enriched within the vRNP-host interaction networks (Fig. 3D and E). These findings suggest a close relationship between AIV infection and biological processes related to host transcriptional regulation and translation mechanisms.

### Functional implication of the vRNP partners

**Fig 4:**
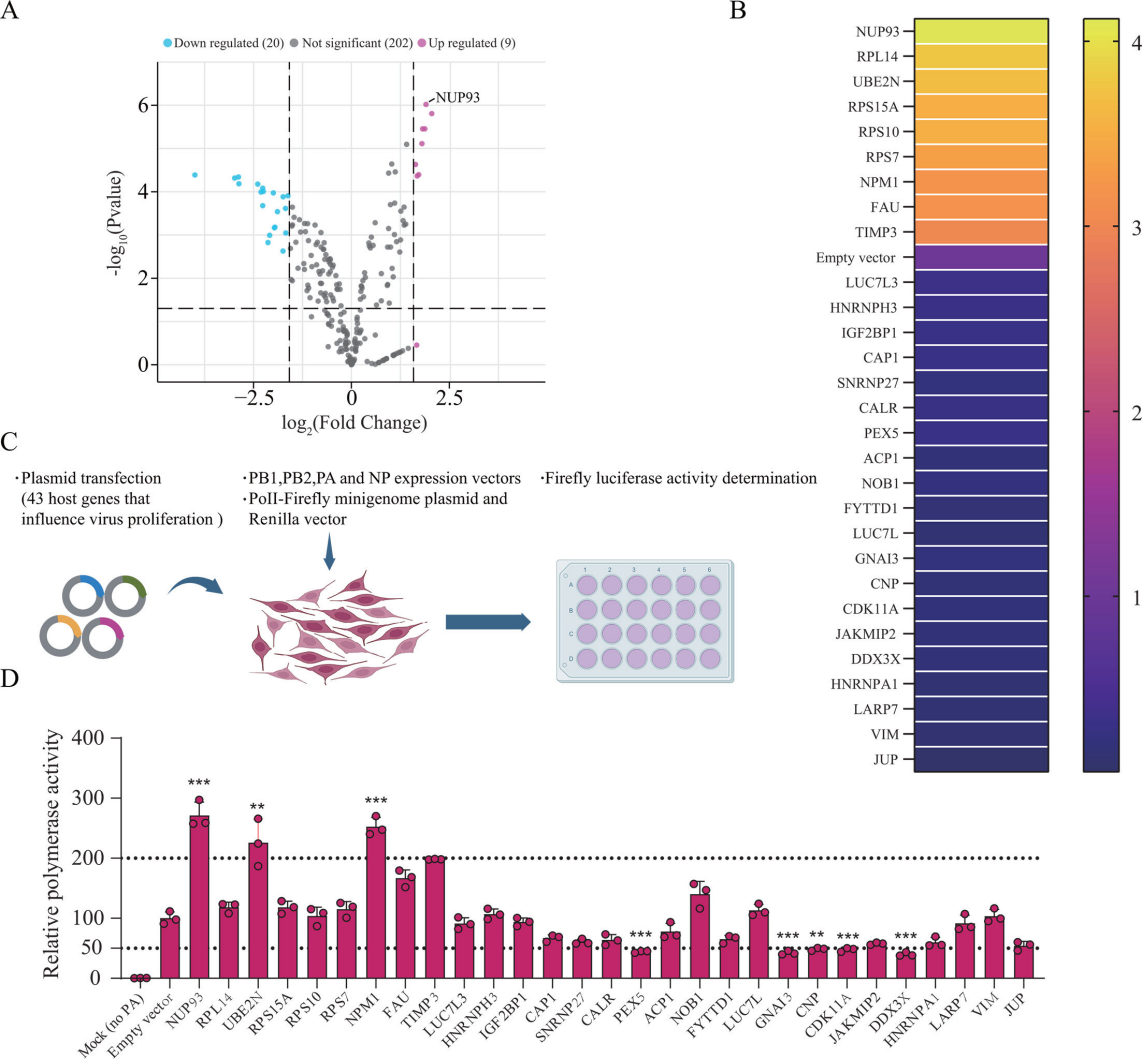
Screening of avian host proteins interacting with vRNP to regulate influenza viral replication. (**A**) The effect of overexpression of 231 host genes in DF-1 cells on influenza virus replication. (**B**) Heatmap of the 29 host genes that significantly regulated AIV replication. Higher rates of GFP positive cells are indicated in yellow, whereas lower rates are indicated in blue, corresponding to a higher degree of pro-viral activity. (**C and D**) Schematic diagram of the minigenome replication assay for assessing the effects of 29 host genes on viral polymerase activity (**C**). Effects of 29 host genes on AIV polymerase activity (**D**). Data were collected from three independent experiments and presented as mean ± SD. Statistical significance was determined using a two-tailed student’s *t*-test. **P < 0.01; ***P < 0.001.

Based on the functional enrichment of host factors targeted by influenza virus polymerase, we conducted experiments based on overexpression to assess the impact of these interacting proteins on the influenza virus life cycle. Among 455 host factors identified by IP/MS, 231 were successfully cloned into the pCMV-3 × FLAG vector. Each candidate host gene plasmid was transfected into the DF-1 cells. After transfection for 24 h, the cells were infected with recombinant H5N6-GFP virus at an MOI of 0.01. The cells were then fixed and analyzed by flow cytometry 24 h after infection. The results showed that 9 positively regulated genes (green fluorescent cell numbers increased by more than 3 times) and 20 negatively regulated genes (green fluorescent cell numbers increased by less than 0.3 times) significantly affected influenza virus replication (Fig. 4A and B), indicating that these host proteins may play an important role in the influenza virus life cycle. Therefore, we hypothesized that these polymerase-interacting partners, affecting viral replication, can specifically modulate viral polymerase activity. This was tested using microgenome replication assays in which each candidate gene was overexpressed. The AIV polymerase subunit and NP genes, viral polymerase RNA template reporter plasmid expressing firefly luciferase, and a plasmid expressing each host gene were co-transfected into DF-1 cells. Luciferase activity was measured after transfection for 24 h (Fig. 4C). The results showed that the eight genes essential for viral replication exhibited significantly altered polymerase activity following overexpression. NUP93, UBE2N, and NPM1 had twice more RdRP transcriptional activity than the control. PEX5, GNAI3, CNP, CDK11A, and DEAD-box helicase 3 X-linked (DDX3X) showed two times lower RdRP transcriptional activity than the control (Fig. 4D). Detailed information on the 231 genes is provided in Table S6. Importantly, NUP93 significantly promoted viral replication and polymerase activity, indicating that it may play a key role in viral polymerase function. Collectively, these avian proteins are the host factors required for AIV polymerase activity, which is a mandatory step in influenza virus replication.

### NUP93 facilitates the proliferation of AIV

**Fig 5:**
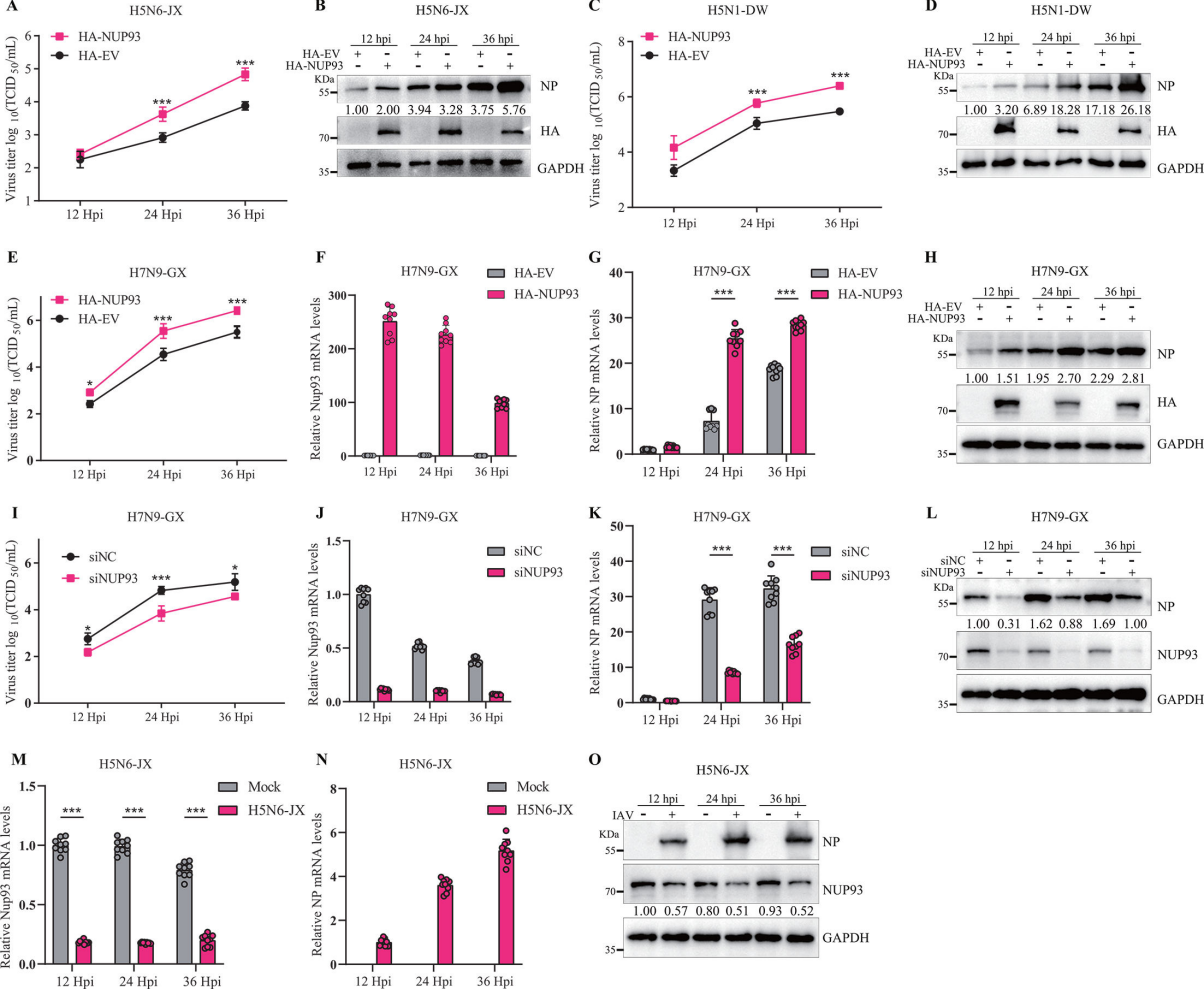
Avian NUP93 facilitates AIV replication in DF-1 cells. (**A through D**) DF-1 cells were transfected with either an empty vector or HA-NUP93 for 24 h, followed by infection with H5N6-JX and H5N1-DW viruses at an MOI of 0.01. Cell supernatants were collected at the indicated time points post-infection and assayed for virus titers using TCID_50_ (panels A and C). The expression levels of NUP93 and viral proteins were analyzed by Western blotting (panels B and D). (**E through L**) DF-1 cells transfected with specified plasmids or siRNAs for 24 h were infected with H7N9-GX virus (MOI = 0.01). At 12, 24, and 36 h post-infection (hpi), virus titers were determined using TCID_50_ (panels E and I); relative mRNA levels of NUP93 and nucleoprotein (NP) were quantified by qPCR (panels F, G, J, and K); and protein levels were evaluated by Western blotting (panels H and L). (**M through O**) DF-1 cells were infected with or without H5N6-JX virus at an MOI of 0.1 for 12, 24, and 36 h. The mRNA and protein levels of NUP93 and NP were measured by qPCR (panel M) and Western blot analysis (panel O). Data are presented as mean ± SD from three or nine independent experiments. Statistical significance was assessed using two-way ANOVA. *P < 0.05; ***P < 0.001.

Given the significant enhancement of influenza virus polymerase activity by NUP93, we initially validated the effect of NUP93 overexpression on wild-type H5N6-JX virus infection. DF-1 cells were transfected with HA-NUP93 or HA-empty vector (HA-EV) for 24 h, followed by infection with the H5N6-JX virus. Culture supernatants were harvested at 12, 24, and 36 h post-infection (hpi), and viral titers were quantified using the 50% tissue culture infective dose (TCID_50_) assay. The results revealed that ectopic expression of NUP93 significantly elevated viral titers compared to the empty vector control (Fig. 5A). To further elucidate the role of NUP93 in influenza A virus (IAV) replication, we performed Western blotting to detect viral NP in infected cells. Notably, NP expression was substantially higher in NUP93-overexpressing DF-1 cells than in control cells (Fig. 5B). To determine whether NUP93 exhibits broad-spectrum proviral activity, we extended our investigation to two additional AIV strains: H5N1-DW and H7N9-GX. Our findings demonstrated that NUP93 overexpression also enhanced the replication of both H5N1-DW and H7N9-GX viruses, as evidenced by the increased viral titers in culture supernatants and elevated levels of viral NP in cell pellets (Fig. 5C through E and H). Furthermore, quantitative analysis of intracellular mRNA levels revealed significantly higher NP mRNA levels in NUP93-overexpressing cells compared to controls (Fig. 5F and G). To further substantiate our hypothesis that NUP93 facilitates IAV propagation, we examined the effects of siRNA-mediated NUP93 knockdown during H7N9-GX virus infection. Viral titers in NUP93-silenced DF-1 cells were markedly reduced at specific time points, consistent with decreases in both viral NP mRNA and protein levels (Fig. 5I through L). Additionally, we explored whether influenza virus infection modulates NUP93 expression. DF-1 cells infected with H5N6-JX virus were analyzed for NUP93 and NP mRNA and protein levels. The results indicated a significant reduction in NUP93 mRNA and protein abundance in H5N6-JX-infected DF-1 cells, which correlated positively with viral replication over the course of infection (Fig. 5M through O). Taken together, these findings underscore the role of NUP93 in promoting the proliferation of diverse AIV strains in DF-1 cells.

### The middle term of NUP93 interacts with PB1 and facilitates AIV replication

**Fig 6:**
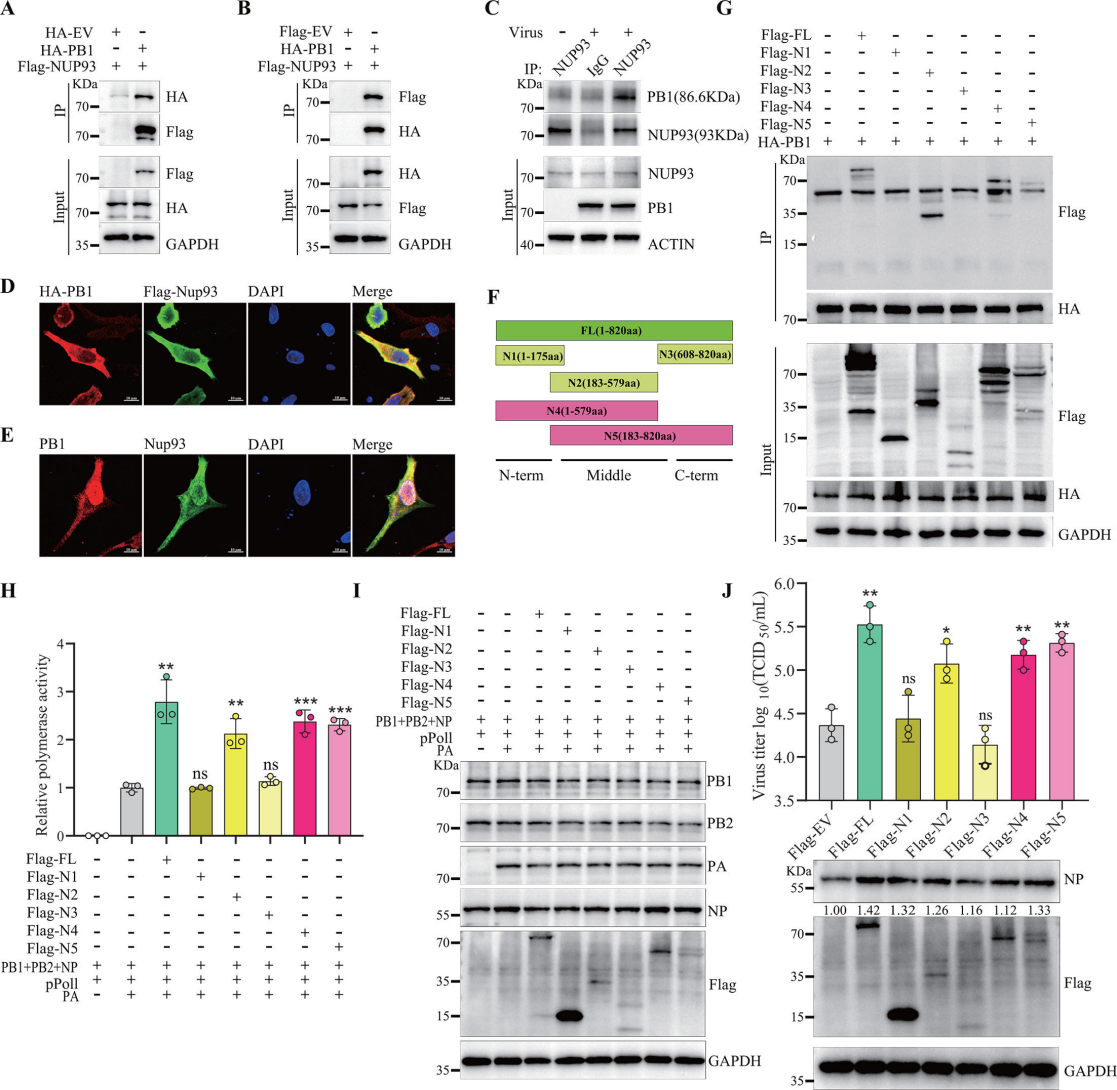
The interplay of avian NUP93 and viral PB1 protein. (**A and B**) DF-1 cells were co-transfected with Flag-NUP93 and HA-PB1. Co-IP experiments were performed using anti-Flag or anti-HA antibodies, followed by Western blot analysis. (**C**) DF-1 cells from 10 cm dishes were infected with H5N6-JX (MOI = 0.01). After 12 h, the cells were lysed with 500 µL IP lysis buffer, and immunoprecipitation was performed using anti-NUP93 or control IgG antibodies. The pull-down products were analyzed using Western blotting. (**D and E**) The localization of exogenous (**D**) or endogenous (**E**) NUP93 (green) and PB1 (red) was assessed via an indirect immunofluorescence assay using confocal microscopy. Scale bar: 10 µm. (**F**) Schematic diagram of the NP truncated segments. (**G**) DF-1 cells in 6-well plates were co-transfected with Flag-EV, Flag-N1, Flag-N2, Flag-N3, Flag-N4, or Flag-N5, along with HA-PB1 using Lipofectamine 2000. At 24 h post-transfection, the cells were lysed, and the co-IP assay was performed using an anti-HA magnetic beads followed by western blot analyses. (**H and I**) DF-1 cells were transfected with plasmids encoding PA, PB1, PB2, NP, and pPolI, along with truncation mutants of NUP93 or an empty vector, as indicated. After 24 h, luciferase activities were measured using a dual-luciferase reporter assay, with *Renilla* luciferase serving as an internal control. (**J**) DF-1 cells were transfected with the indicated NUP93 truncation mutants for 24 h, followed by infection with H5N6-JX virus at an MOI of 0.01. Cell supernatants and lysates were harvested at 24 hpi for TCID_50_ assays and western blot analysis. Data were collected from three independent experiments and are presented as mean ± SD. Statistical significance was determined using a two-tailed student’s *t*-test. ns, not significant; *P < 0.05; **P < 0.01; ***P < 0.001.

NUP93 significantly enhanced the proliferation of influenza virus, and the AP-MS data revealed a high-confidence interaction between NUP93 and the viral protein, PB1 (Table S1). To further investigate the association between PB1 and NUP93, we performed co-immunoprecipitation (Co-IP) assays using the DF-1 cell transfection system. The results demonstrated that HA-tagged PB1 could be co-immunoprecipitated with Flag-tagged NUP93 (Fig. 6A). Reciprocally, Flag-NUP93 was co-precipitated using anti-HA magnetic beads in the presence of HA-PB1 (Fig. 6B). Additionally, endogenous NUP93 and PB1 were co-precipitated with anti-NUP93 antibody in H5N6-JX-infected DF-1 cells at 12 hpi (Fig. 6C). Given the interaction between NUP93 and PB1, co-localization between the interacting partners should be examined by immunofluorescent staining. To examine their co-localization, Flag-NUP93 and HA-PB1 were co-transfected into DF-1 cells. The results revealed that NUP93 was located in the cytoplasm and nucleus of the cells, and co-localization was detected in the cytoplasm (Fig. 6D). Similar patterns were observed in H5N6-JX-infected DF-1 cells (Fig. 6E). To delineate the interaction mechanism, we constructed various NUP93 truncation mutants: N-terminal (aa 1–175), middle region (aa 183–579), C-terminal (aa 608–820), and deletion mutants lacking either N- or C-terminus (aa 183–820 and aa 1–579, respectively) (Fig. 6F). Co-IP assays performed 24 h post-transfection confirmed successful expression of all truncations, but only the middle region (aa 183–579) was precipitated by HA-tagged PB1 (Fig. 6G). Subsequent evaluation of AIV polymerase activity revealed that full-length of NUP93 (FL, aa 1–820), N2 (aa 183–820), N4 (aa 1–579), and N5 (aa 183–820), and all enhanced the relative polymerase activity (Fig. 6H). Importantly, overexpression of these truncators did not affect expression levels of PA, PB1, PB2, or NP (Fig. 6I), indicating that the truncators containing the middle region (aa 183–579) specifically enhanced the polymerase activity without altering RNP component expression. Further experiments showed that transfection of the NUP93 middle region (aa 183–579) into DF-1 cells followed by H5N6-JX infection significantly promoted viral production, as quantified by viral titers in culture supernatants and viral NP levels in cell lysates (Fig. 6J). Which is consistent with the results of the polymerase activity assay. Collectively, these findings demonstrate that the middle region (aa 183–579) of avian NUP93 interacts with PB1 and facilitates viral replication by enhancing polymerase activity.

## DISCUSSION

For decades, various techniques have been used to characterize the interaction networks between influenza viruses and host proteins, including AP-MS and yeast two-hybrid (Y2H) (13, 27). Currently, AP-MS is more widely used than Y2H because it can reveal simulated interactions in cells under physiological conditions. Furthermore, several recent studies focused on identifying interaction factors between influenza viruses and human hosts to better understand their pathogenesis and infection mechanisms. However, there are few reports on the interactions between AIVs and avian host proteins, especially concerning the H5N6 strain. In this study, we used IP-coupled liquid chromatography-mass spectrometry (LC-MS)/MS technology to identify host factors that interact with the H5N6-JX influenza virus polymerase in DF-1 cells. By transfecting the virus polymerase plasmid and directly infecting the cells, we identified 455 host factors potentially interacting with the viral polymerase. Among these, 421 were previously implicated in IAV infection, underscoring the evolutionary conservation of these genes’ functions across avian and mammalian species. Notably, we discovered 34 novel candidate genes that had not been previously associated with IAV replication. The potential roles of their mammalian orthologs in IAV proliferation warrant further investigation. We discovered that the host proteins identified in this study are primarily involved in peptide metabolic processes, translation, and actin filament binding, which are closely related to viral replication and pathogenesis. Furthermore, we confirmed the involvement of several avian host proteins in the replication process of the influenza virus through gene overexpression and polymerase activity experiments. This indicates that many avian host factors interact with the polymerase complex and are essential for viral genome transcription and replication.

The viral RNA polymerase is composed of PA, PB1, PB2, and NP. PB1 is the core subunit of the complex containing polymerase activity, whereas PB2 recognizes capped cellular mRNA (28), and PA has endonuclease activity (29). During the IAV life cycle, the vRNPs enter the host cell and are transported to the nucleus for replication. In the cell nucleus, polymerases also allow the genome to be transcribed into mRNA, which is then transported back into the cytoplasm and translated into viral proteins. NP, PB1, PB2, and PA proteins then reenter the cell nucleus to form RNP complexes with viral RNA (17). KEGG pathway enrichment analysis of the polymerase and host interaction factors of the H5N6 AIV revealed significant enrichment in the ribosome pathway, represented by RPL17, RPLP2, and RPS29. An important future direction for this work is to provide a detailed explanation of how these ribosomal proteins affect influenza virus replication. The ribosome protein (RPL) interacts extensively with AIV and plays a crucial role in the adaptation of the virus to its host (30–32). Several RPL proteins, including RPL17, promote the interaction between hepatitis C virus RNA and core protein and promote the assembly of virus particles (33). RPLP2 interacts with eIF4E through binding to the 5′ UTR of porcine epidemic diarrhea virus, and RPLP1 occupies the C/EBPβ binding site in the viral long terminal repeat to inhibit HIV-1 transcription (34). RPL18 interacts with the nonstructural protein of Dengue virus (DENV) and significantly reduces virus translation, replication, and viral yield, indicating that RPL18 is essential for the replication cycle of DENV (35). Additionally, the spliceosomes, basal transcription factors, and nucleocytoplasmic transport pathways may play important roles in IAV infection. Therefore, we hypothesized that research on these pathways might provide a good approach for studying the pathogenesis of IAV infections.

In this study, we successfully cloned 231 of 455 potentially interacting proteins from avian host genes. After overexpressing these genes in DF-1 cells and analyzing them by flow cytometry, we found that 20 genes could inhibit the replication of AIV (with a 3-fold change). These genes included LUC7L3, HNRNPH3, IGF2BP1, and CAP1. Among these host genes, the host protein DDX3X is crucial for coordinating multifaceted innate antiviral responses during IAV infection. It inhibits the proliferation of influenza viruses by regulating the activation of the nucleotide-binding oligomerization domain-like receptor and pyrin domain 3 inflammasome, assembly of stress granules, and type I interferon (IFN) response (36). Human hnRNPA1 interacts with NP to inhibit the proliferation of H1N1-PR8 in A549 cells (37), which is consistent with our findings in avian DF-1 cells. Additionally, the absence of vimentin in MEFs inhibits endosome trafficking and acidification, significantly reducing the expression of influenza viral RNA and proteins and the production of infectious progeny viruses (38). In addition, nine genes were identified to significantly promote the proliferation of the AIV (with a threefold change), including NUP93, RPL14, UBE2N, and RPS15A. NPM1 enhances the activity of influenza virus H5N1 polymerase and promotes viral replication (39). NPM1 can reportedly promote viral replication through direct interaction with the circovirus cap protein (Cap) (40). Human NUP93 facilitates influenza virus replication by regulating the nuclear export of viral RNA (41). In this study, the interacting proteins of the influenza virus polymerase that we screened influenced the proliferation of the influenza virus by modulating the host’s innate immunity and regulating the transcriptional and replicative activities of the viral polymerase, indicating the importance of these host factors in the influenza virus life cycle. Furthermore, because of the significant promotion of influenza virus polymerase activity by NUP93 in DF-1 cells, we conducted more in-depth studies on the interaction partners of influenza virus polymerase.

After the influenza virus infects the cells, vRNPs must be transported to the nucleus through the nuclear pore complex (NPC), where viral genome transcription and replication occur. NUP93 is an “on-pore” nucleoporin that functions as a chromatin anchor during differentiation (42). NUP93 mutations also eliminate protein-protein interactions between SMAD4 and importin 7, potentially disrupting nucleocytoplasmic transport. Additionally, severe acute respiratory syndrome coronavirus nonstructural protein 1 (Nsp1) reportedly disrupts the localization of NUP93 around the nuclear envelope. Nsp1 can regulate multiple steps in gene expression, including nucleocytoplasmic transport (43). This suggests a close association between NUP93 and viral infections. Furthermore, recent studies have shown that the knockdown of human NUP93 in A549 cells reduces viral replication and is involved in the nuclear export of viral RNA (41), indicating NUP93 as an important host factor for influenza virus infection. However, the interaction between NUP93 and influenza virus polymerase has not been previously reported. In this study, we demonstrated that NUP93 overexpression in DF-1 cells significantly promoted the proliferation of the AIV H5N6-JX, H5N1-DW, and H7N9-GX, whereas NUP93 knockdown inhibited AIV proliferation. These results are consistent with those obtained from human A549 cells. Previous studies have shown that a reduction in NUP93 expression can affect the proper assembly of NPC (44, 45), and NUP93 plays a crucial role in retinoic acid-inducible gene I-like receptor-mediated antiviral responses (46). Interestingly, we observed a significant decrease in NUP93 expression in DF-1 cells infected with influenza virus, which may be due to the combined effects of the host and virus on the NUP93 regulation of viral replication by modulating NPC assembly and innate immune responses. These results strongly suggest an important NUP93 role in influenza infection. However, the mechanism underlying the downregulation of NUP93 expression during influenza infection remains unclear and requires further investigation. Based on our AP-MS data, we identified NUP93 as a high-confidence interacting partner of the viral polymerase subunit, PB1. Furthermore, we confirmed the interaction between NUP93 and the PB1 subunit using co-immunoprecipitation in an exogenous plasmid transfection system. Additionally, in DF-1 cells infected with H5N6-JX, PB1 was immunoprecipitated by endogenous NUP93, suggesting a critical role for NUP93 during viral infection. Previous reports have indicated that human NUP93 not only facilitates the nuclear export of viral RNA but also promotes the nuclear export of host mRNA. We found that avian NUP93 significantly enhances the activity of AIV polymerase, leading us to speculate that avian NUP93 may also promote the nucleocytoplasmic translocation of AIV polymerase proteins or RNA, thereby facilitating viral replication.

In conclusion, through exogenous and endogenous immunoprecipitation experiments, we constructed an interaction map of the H5N6 polymerase with chicken proteins. We successfully cloned 231 out of 455 host factors for meticulous overexpression analysis in DF-1 cells. We identified 9 avian genes with the potential to enhance viral replication and an additional 20 genes with a significant inhibitory effect on viral proliferation. Notably, we discovered that avian NUP93 interacts with the AIV PB1 protein and promotes the replication of the H5N6 AIV by enhancing the activity of the AIV polymerase. In summary, these findings emphasize the significance of NUP93 as an interacting factor in viral replication.

## Data Availability

The processed mass spectrometry data sets generated during this study are available from the corresponding author upon reasonable request. All other data supporting the findings are included in this article and its supplemental files.

## SUPPLEMENTAL MATERIAL

Supplemental figures spectrum.03120-24-s0001.jpg Determination of infection and transfection conditions in DF-1 cells.

Project Completion Report spectrum.03120-24-s0002.docx Detailed documentation of the experimental procedures and quality controls.

Search Results spectrum.03120-24-s0003.xlsx All processed MS data, including experimental vs. control group comparisons.

Table S1 spectrum.03120-24-s0004.xlsx Identified host proteins interacting with vRNP.

Table S2 spectrum.03120-24-s0005.xlsx Primers and small interfering RNAs used in this study.

Table S3 spectrum.03120-24-s0006.xlsx Comparison of host factors identified in this study with those identified in the indicated studies.

Table S4 spectrum.03120-24-s0007.xlsx The GO functional enrichment analyses of host proteins interacting with virus.

Table S5 spectrum.03120-24-s0008.xlsx The KEGG functional enrichment analyses of host proteins interacting with virus.

Table S6 spectrum.03120-24-s0009.xlsx The results of flow cytometric analysis of 231 host genes.

ASM does not own the copyrights to Supplemental Material that may be linked to, or accessed through, an article. The authors have granted ASM a non-exclusive, world-wide license to publish the Supplemental Material files. Please contact the corresponding author directly for reuse.
