# Supplementary figures and images for: Viral polymerase-host interaction analysis reveals that the association between avian NUP93 and PB1 promotes H5N6 avian influenza virus replication
Source: Microbiol Spectr. 2025 May 23;13(7):e03120-24. doi: 10.1128/spectrum.03120-24 (PMC12211069; doi:10.1128/spectrum.03120-24)

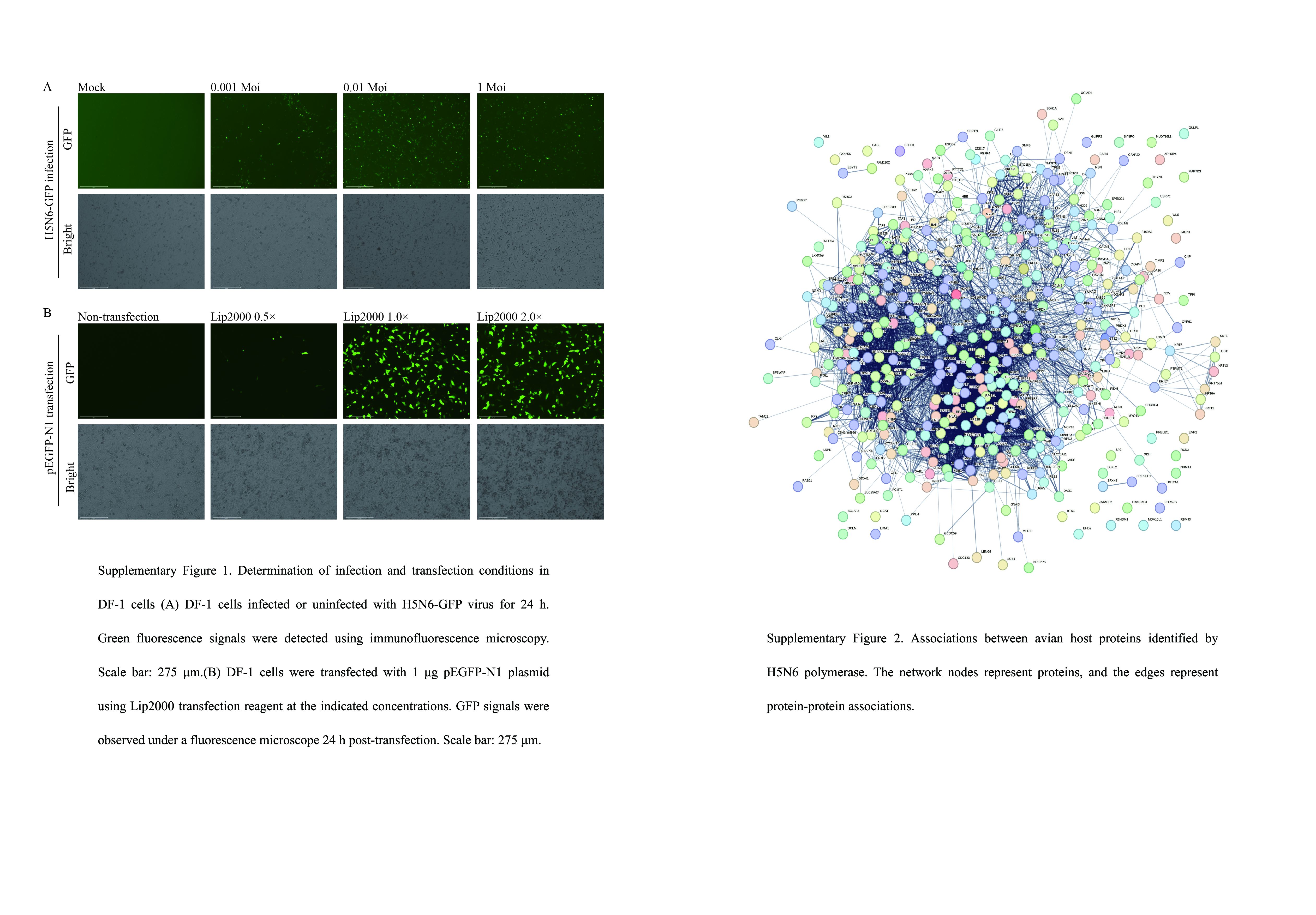

Supplement: Supplemental figures — Determination of infection and transfection conditions in DF-1 cells. [file spectrum.03120-24-s0001.jpg]
