# Supplementary material for: Viral polymerase-host interaction analysis reveals that the association between avian NUP93 and PB1 promotes H5N6 avian influenza virus replication
Source: Microbiol Spectr. 2025 May 23;13(7):e03120-24. doi: 10.1128/spectrum.03120-24 (PMC12211069; doi:10.1128/spectrum.03120-24)
Supplement: Project Completion Report — Detailed documentation of the experimental procedures and quality controls. [file spectrum.03120-24-s0002.docx]

Dear Editor,

This is the completion report of the mass spectrometry project from that time, primarily conducted in 2019. It documents detailed information about the experiments conducted, as well as mass spectrometry base peak charts. The pictures can be enlarged.

PB2 and NP

PB1

Virus-PB1
